# Modeling, Control, and Numerical Simulations of a Novel Binary-Controlled Variable Stiffness Actuator (BcVSA)

**DOI:** 10.3389/frobt.2018.00068

**Published:** 2018-06-15

**Authors:** Irfan Hussain, Ahmad Albalasie, Mohammad I. Awad, Lakmal Seneviratne, Dongming Gan

**Affiliations:** ^1^Khalifa University Center for Autonomous Robotic Systems (KUCARS), Khalifa University of Science and Technology, Abu Dhabi, United Arab Emirates; ^2^Department of Mechanical and Mechatronics Engineering, Birzeit University, Birzeit West Bank, Palestine

**Keywords:** variable stiffness actuators, MPC, multiple MPC, safety, human-robotic interaction, actuator control

## Abstract

This research work aims at realizing a new compliant robotic actuator for safe human-robotic interaction. In this paper, we present the modeling, control, and numerical simulations of a novel Binary-Controlled Variable Stiffness Actuator (BcVSA) aiming to be used for the development of a novel compliant robotic manipulator. BcVSA is the proof of concept of the active revolute joint with the variable recruitment of series-parallel elastic elements. We briefly recall the basic design principle which is based on a stiffness varying mechanism consisting of a motor, three inline clutches, and three torsional springs with stiffness values (***K***_**0**_, 2***K***_**0**_, 4***K***_**0**_) connected to the load shaft and the motor shaft through two planetary sun gear trains with ratios (4:1, 4:1 respectively). We present the design concept, stiffness and dynamic modeling, and control of our BcVSA. We implemented three kinds of Multiple Model Predictive Control (MPC) to control our actuator. The main motivation of choosing this controller lies in the fact that working principle of multiple MPC and multiple states space representation (stiffness level) of our actuator share similar interests. In particular, we implemented Multiple MPC, Multiple Explicit MPC, and Approximated Multiple Explicit MPC. Numerical simulations are performed in order to evaluate their effectiveness for the future experiments on the prototype of our actuator. The simulation results showed that the Multiple MPC, and the Multiple Explicit MPC have similar results from the robustness point of view. On the other hand, the robustness performance of Approximated Multiple Explicit MPC is not good as compared to other controllers but it works in the offline framework while having the capability to compute the sub-optimal results. We also performed the comparison of MPC based controllers with the Computed Torque Control (CTC), and Linear Quadratic Regulator (LQR). In future, we are planning to test the presented approach on the hardware prototype of our actuator.

## Introduction

Several aspects and design requirement have been raised to enhance the quality of human-robot interaction and co-work collaborations in smart manufacturing and domestic scenarios (Bicchi et al., 2008; Tsagarakis et al., 2011; Gan et al., 2012; Asota et al., 2017). Safety is considered one of those most important aspects, as well as the general aspects of efficiency and robustness which are taken with high importance. Safety should be an intrinsic feature in robots especially in the case of unexpected interactions, or sensor failures (Asota et al., 2017). Besides the safety aspect, the interaction between the robot and the operator must show adaptability and force accuracy (Hussain et al., 2016; Salvietti et al., 2017b).

Intrinsic compliance introduced in robotic systems is the one of possible solution toward the robustness and safe human-robotic interaction (Hussain et al., 2017; Salvietti et al., 2017a). Bio-inspired robotics design results in compliant robotic systems with improvement natural dynamics and kinematics performance (Hogan, 1985; Migliore et al., 2005; Shin et al., 2010). These improvements have led to the development of variable impedance actuators (VIA), of which the actuator mechanical properties (inertia, damping, or stiffness) affect the system equilibrium position (Bicchi et al., 2008). This alters the interaction forces in order to adapt the different situations between the robots and the environment/users, leading to safer energy efficient operations (Bicchi et al., 2008).

Based on how the impedance (stiffness and damping) is achieved, both active and passive VIA concepts were proposed in the literature. In a feature in active-by-control lies in its ability to adapt both stiffness and damping in a wide range and for several speeds. The disadvantages of this system lie in the need of very accurate and expensive force/torque sensors, the high energy consumption, the need of a complex control system, the incapability of energy storage and shock absorption (Hogan, 1985; Mayne, 2014). In order to solve these problems, passive compliant elements can be added to the actuator. An early approach was proposed in the Serial Elastic Actuator (SEA) (Shin et al., 2010). Several techniques on the SEA structures and control systems were presented in the literature (Migliore et al., 2005; Bischoff et al., 2010). The drawbacks of the SEA lie in the non-optimal performance and non-optimal energy efficiency. The optimal performance needs careful tuning of the joint stiffness values (Pratt and Williamson, 1995). The SEA based actuator stiffness is fixed and determined by the spring selection, thus the physical stiffness cannot be changed during operation.

This motivated lots of study and new designs of variable stiffness mechanisms with passive compliance (Tonietti et al., 2005). Several groups have designed adaptable compliance mechanisms, with elastic elements storing energy, in addition to altering the stiffness.

Many topologies and realization for Variable Stiffness Actuators (VSA) were presented in the literature. The initial evolution was the concept of the antagonistic variable stiffness actuators, where the joint stiffness is varied through the combination of two antagonistic SEAs controlled by two separate motors. Designs which fall into this category include VSA-I (Tonietti et al., 2005), VSA-II (Schiavi et al., 2008), AMASC (Hurst et al., 2010), and the biological inspired joint stiffness control mechanism (Migliore et al., 2005). Moreover, another realization for stiffness altering is achieved through the principle of the lever mechanism (Sun et al., 2017). Other examples of this type include the AwAS (Jafari et al., 2010), AwAS-II (Jafari et al., 2011), the vsaUT (Visser et al., 2011), the mVSA-UT (Fumagalli et al., 2012), the vsaUT-II (Groothuis et al., 2014; Guo et al., 2017), and pVSJ (Awad et al., 2016a).

Although, the VSA give intrinsic capabilities (bandwidth, impacts, energy storage) over the joint stiffness range and overcomes the limitation of SEA by allowing the stiffness variation continuously but need more energy consumption. In particular, two motors are required: one to control the equilibrium position and the second to control stiffness.

Lately, a new approach in varying the stiffness is followed through discretely selecting the level of stiffness by adding/subtracting the number of involved elastic elements. The elastic elements are engaged through a locking mechanism or simply a clutch. The elastic elements can either be arranged in series, like the pDVSJ (Awad et al., 2016b), or in parallel. A realization of the later arrangement is the Series-Parallel Elastic Actuators (SPEA).

The driver for designing the Series-Parallel Elastic Actuators (SPEA) was the need to minimize energy consumption, peak torque, or power consumption in robots and humans (Mathijssen et al., 2015b). This concept is introduced in the iSPEA (Mathijssen et al., 2016). In this actuator, the springs are connected to the output link from one side and to an intermittent mechanism on the other side. This mechanism converts a continuous (rotational) input into two consecutive phases.

This yields a succession of springs' involvement by altering the output torque of the actuator. MACCEPA-Based SPEA is another example of SPEA and is illustrated in Mathijssen et al. (2015a), where the springs are recruited through a cylindrical cam mechanism. Another illustration is the +SPEA (Mathijssen et al., 2016).

The stiffness is altered by changing the number of involved torsional springs by the activation/de-activation of clutches. The clutches used in BpVSJ are Electromagnetic (EM) clutches (Huco, SO 17) which operate electrically but transmit torque mechanically. The difference between electromagnetic clutch and the regular clutch is in how they control the movement of pressure plates. In the normal clutch, a spring used to engage the clutch whereas in EM clutch an electromagnetic field is used for engagement. The main components of EM clutch are a coil shell, an armature, rotor, and hub. The armature plate is lined with friction coating. The coil is placed behind the rotor. When the clutch activated the electric circuit energizes the coil, it generates a magnetic field. The rotor portion of clutch gets magnetized. When the magnetic field exceeds the air gap between rotor and armature and then it pulls the armature toward the rotor. The frictional force generated at the contact surface transfer the torque. When voltage is removed from the coil the contact is disengaged.

**Figure 1:**
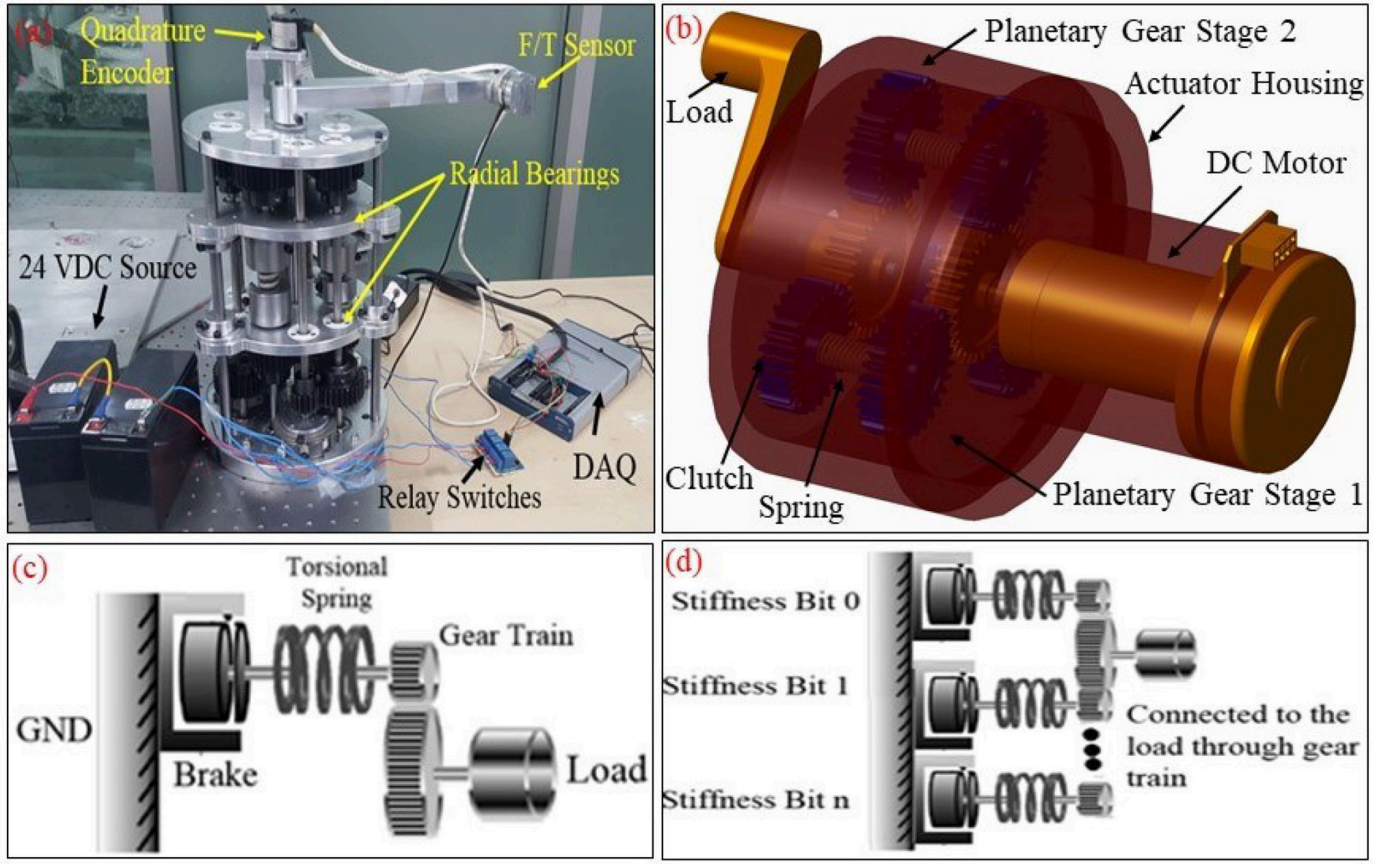
**(a)** Prototype of the passive version of the actuator (Passive Binary Controlled Variable Stiffness Joint (BpVSJ) **(b)**. CAD model of the binary-controlled variable stiffness actuator (BcVSA): It is the active and compact version of the passive joint **(c)**. The conceptual diagram for the working principle of the BcVSA. Stiffness Bit, the set of a grounded brake connected to an elastic element (spring) which would react against the load torque through the gear train. **(d)** Multiple Stiffness Bits connected to the output link (Load) through combined gear trains.

The design of the actuator proposed in this paper is inspired by its passive version (BpVSJ) (Awad, 2018), see Figure 1a, having similar working principle but compact in size and having a motor to control it actively. The CAD model of the actuator is shown in Figure 1b and we named it, binary-controlled variable stiffness actuator (BcVSA). The additive advantage of this topology allows more freedom in selecting the level of stiffness without the need of succession involvement, leading to lesser response time in altering the stiffness level. We believe that this novel topology can be applied in BcVSA for a safer, more efficient, human-robot complaint manipulator. The conceptual diagram of the proposed actuator is shown in Figures 1c,d while the detailed explanation and stiffness modeling is presented in section Concept and Stiffness Model.

Another challenging task is the control system design of robotic actuators. In literature, several approaches (with their own pros and cons) to control Variable Stiffness Actuators are presented. Most of them are based on Non-linear controllers. Some of the examples are, Gain scheduling through LQR (Sardellitti et al., 2013), Sliding Mode Controller (Van Damme et al., 2009), Output-Based Controller (Palli and Melchiorri, 2011), and neural network based controller (Guo et al., 2017; Pan et al., 2017) etc.

In this paper, we propose multiple MPC to control our actuator. The main motivation of choosing this controller lies in the fact that working principle of multiple MPC and multiple states space representation of our actuator fit to each other very well. As a matter of fact, our system has multiple levels of stiffness (state space representations) and Multiple MPC has the capability to provide the optimal solution for each state (having dedicated MPC). As examples, we also implemented the Computed Torque Control (CTC), and Linear Quadratic Regulator (LQR) to perform the comparison with Multiple MPC based controllers. We have used three controllers from the family of Multiple MPC for our actuator. MPC is categorized as an optimal discrete controller as it solves a quadratic programming (QP) problem to compute the optimal or the sub-optimal response for the system. On the other hand; the QP is classified as a constrained optimization problem where the cost function is selected based on the design requirements. Common design requirements are related to minimizing the dynamic errors to increase the accuracy and the precision of the system, also the energy consumption i.e., the input signals to maximize the energy saving, and the change rates of the input signal (Albalasia, 2016). Based on that, the primary structure of MPC consists of an optimizer to solve the QP problem, a model of the plant and an observor to estimate the unmeasured states using a steady-state Kalman filter and the observor is used also to predict the behavior of the plant in a specific time in advanced (Matlab, 2018). While the target of the optimizer is to minimize the cost function with respect to the constraints.

As detailed in Camacho and Alba (1999), MPC has three essential strategies to compute the optimal control. These strategies are summarized in the followings:

- Predict the response of the system for a specific time. Usually this time is called a prediction horizon (P) and this strategy is performed by using an observer (Matlab, 2018).
- MPC solves the QP problem to compute the control actions (C) (MPC can compute more than one actions) and this is achieved by solving the reduced form for the algebraic Riccati equation.
- MPC utilizes only the current control action C(k) to avoid any unexpected behavior for the system in the future due to a measured or unmeasured disturbance on the system. It means the response may change if the control is not robust.

These strategies are working continuously to update the behavior of the system and to compute the correct control actions C(k). According to Camacho and Alba (1999), MPC has several features from a control point of view e.g., it has the ability of disturbance rejection for a measured and unmeasured disturbance. Furthermore, it has the ability to take into considerations the physical capability for each actuator (Castillo et al., 2007), On the contrary, the major limitations for this class of control are related to the applicability to use it in real time. Firstly, because it's computationally expensive and controller solves an optimization problem and computes a finite control horizon (C) with respect to the mathematical model for the system with applied constraints. However, this problem can partially be solved by utilizing a microcontroller or computers with high computation capabilities. Secondly, this class of controllers suffers from the feasibility problem because it solves QP problem and there could be a possibility that the optimizer would not find an optimal solution especially in case of hard constraints (Mayne, 2014). However, the suggested solution for this problem is to modify the system itself or to relax the selected constraint as shown in the literature (Albalasia, 2016; Matlab, 2018).

The Explicit MPC and Explicit Multiple MPC are the types of MPC which simulate the system in the off-line framework to calculate the control action then all the solutions are saved in a look-up table i.e., a “database” and multiple affine functions are used to compute the required control action in the online environment (Fiacchini et al., 2006; Matlab, 2018). While the main difference between these two types is that the Explicit MPC can only be used for one equilibrium point while the Explicit Multiple MPC can be used at several equilibrium points i.e., it has a wider range (Matlab, 2018).

Recently, various types of MPCs are used in several applications e.g., linear MPC is used to control the unmanned helicopter and the marine surface vessels as shown in Castillo et al. (2007) and Oh and Sun (2010). However, multiple autonomous surface vessels are controlled using NMPC see the literature (Fahimi, 2007). In addition; Adaptive MPC is utilized to control the under-actuated robot to minimize the energy consumption (Albalasia, 2016; Albalasie et al., 2016). Furthermore, Fia et al. suggested a control scheme for controlling a linear motor by using an Explicit MPC (Fiacchini et al., 2006). While Kuure-Kinsey et al. utilized Multiple MPC to regulate the response of non-linear and uncertain process systems like Van de Vusse reactor as a case study (Kuure-Kinsey and Bequette, 2009). We also performed the comparison of Multiple MPC based controllers with the Computed Torque Control (CTC), and Linear Quadratic Regulator (LQR). The first method is built on a state feedback tracking system using Linear Quadratic Regulator (LQR). controller, detailed in Ogata (2009). On the other hand, the second control scheme is a Computed Torque Control (CTC) scheme, which is classified as model-based control. An examples in literature, where CTC is implemented is, for controlling a PUMA-560 robot manipulator (Piltan et al., 2012). The CTC uses the exact feedback linearization to linearize the nonlinear model if it exists and it converts the dynamic equations of the system to a unit mass system equation but with “n” DOFs. Nevertheless, if the system is classified as an underactuated system this method uses the partial feedback linearization technique to be able to control the plant or the system as shown in Spong (1994).

The rest of the paper is organized as it follows. The conceptual design and the stiffness modeling of the proposed actuator are presented in section Concept and Stiffness Model. Section The Dynamic Modeling of the BcVSA describes the dynamic model of the BcVSA. Section Control Schemes presents the control schemes based on three Multiple MPC control approaches. The numerical simulations are detailed in section Numerical Results. Finally, the conclusions and future work are illustrated in section Conclusions and Future Work.

## Concept and stiffness model

### Concept of BcVSJ

**Figure 2:**
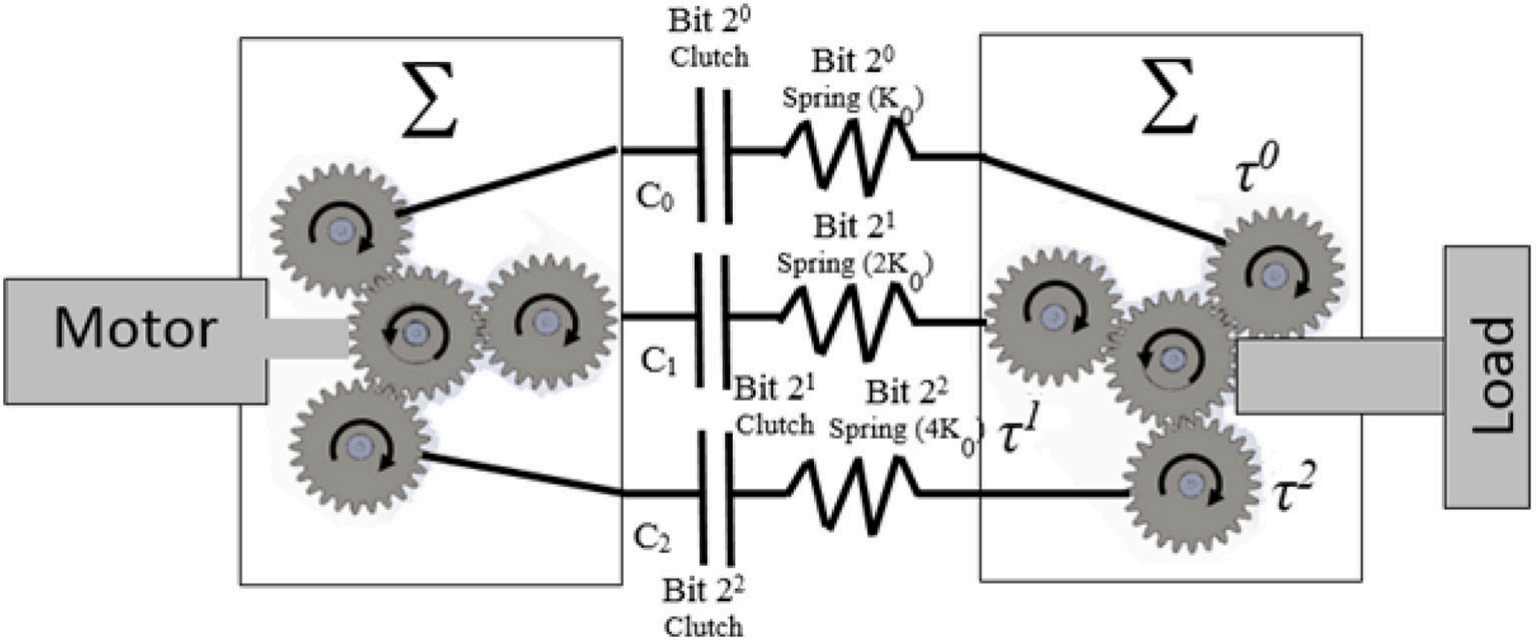
Concept of BcVSA; the stiffness is altered by changing the number of involved springs. Stiffness Model the K_0_ is the base value for the springs and C_0_, C_1_, C_2_ represents clutch,0, 1, 2 respectively.

The designs proposed in the literature about Series Parallel Elastic Actuators was the main inspiration behind the proposed design in this paper. In this type of actuators, the stiffness is altered by changing the number of involved elastic elements. The elastic elements are parallel with respect to each other, and their combined stiffness is connected in series with a load (output link) in one end. The other end can be connected in series with clutching mechanism and an actuator (motor) in an active system as shown Figure 1b. A motor is connected to a spur gear (sun gear) which is engaged with another spur gear (planet gear). An inline clutch is serially connected to a torsion spring and then another spur gear (planet gear) which is then coupled to the main spur gear (sun gear) of the load shaft as in Figure 2.

When the clutch is engaged (active), the power transmission goes through the spring to the load. The planet gear -clutch-torsion spring- planet gear unit is called a Stiffness Bit (see Figure 1c), which provides the stiffness of the torsion spring to the main shaft of the load. By connecting n stiffness bits to the sun gear of the load shaft in parallel, the sum of all the torsion springs stiffness will be the output stiffness of the joint as in Figure 1d. Theoretically, without considering overlapping stiffness results, there are (n+1) different combinations of stiffness by selecting the stiffness bits. Here 1 means the zero stiffness which is omitted in the real case as it is undesirable to totally disengage the load from the motor's grip.

The novelty of the BcVSA lies in the design topology as in Figure 2. Three custom-made torsional springs with three different values (***K***_**0**_, 2 ***K***_**0**_, 4 ***K***_**0**_) respectively are serially connected to inline clutches, which are connected to the motor through sun- planet gears. Each set of (spring-clutch) represents a (Stiffness Bit) in the binary representation of stiffness level. The other end of each spring is connected to a planetary gear which contributes part of the resultant torque on the other sun-gear. The sun gear is connected to the output arm. A stiffness bit transmits energy to the output arm if the bit's clutch is active. If the bit's clutch is inactive, the spring will run-freely. The levels of stiffness that can be represented in binary representation are shown in Table 1. An active stiffness is represented by “1” while an inactive stiffness-bit is represented by “0”. The use of the clutches facilitates fast switching among the stiffness levels. Lastly, the scalability can be achieved either by changing the value of the (***K***_**0**_) or by adding extra Stiffness-Bits.

**Table 1:** Achievable Stiffness Levels of BcVSA.

**Stiffness bit 2^2^(4K_0_)**	**Stiffness bit 2^1^ (2K_0_)**	**Stiffness bit 2^0^ (K_0_)**	**Stiffness level**
0	0	0	0
0	0	1	K_0_
0	1	0	2 K_0_
0	1	1	3 K_0_
1	0	0	4 K_0_
1	0	1	5 K_0_
1	1	0	6 K_0_
1	1	1	7 K_0_

### Stiffness model

The stiffness of BcVSA is altered through changing the number of involved parallel elastic elements. The involvement of an elastic element is achieved through activating the associated clutch connected to the designated spring. The stiffness model will be derived from the kinematics model of the joint illustrated in Figure 2. If the motor's shaft is grounded, and an external torque (τ^Σ^) is exerted on the output shaft, the output shaft will rotate the sun gear. In the case where all stiffness-bits are inactive, the torque and motion will be transmitted freely through the planetary gears, into the torsional springs which they would rotate freely with no compression. In case of any active stiffness-bit(s), the motion of shaft connecting the end of the involved torsional spring to the clutch will be blocked. In the presence of the exerted torque on the output shaft, the involved torsional springs will deflect, yielding a counter torque that would be felt as resistance force by the user's hand.

Deriving the stiffness model starting from the resultant torque (τ^Σ^) equation as follows

(1) $$\begin{array}{r} \tau^{\Sigma }=-\left(\tau^{0}+\tau^{1}+\tau^{2}\right) \end{array}$$

where τ^0^, τ^1^, τ^2^are the torque of the stiffness bits 0, 1, and 2 respectively.

Each of these torques can be represented in the following equation:

(2) $$\begin{array}{r} \tau^{\text{n}}={N_{n}\beta }_{n}\left(2^{n}\left(K_{0}\right)\;\left(\theta -\phi_{n}-\varphi \right)\right)\;,\;n\in \left\{0,1,2\right\} \end{array}$$

(3) $$\begin{array}{r} \phi_{n}=\theta \left(t_{ON,n}\right)\;,\;n\in \left\{0,1,2\right\} \end{array}$$

(4) $$\beta_{n}=\{\begin{array}{c} 0,\\ 1, \end{array}\begin{array}{c} \;if\;Clutch\;\left(n\right)\;is\;inactive\;\left(deactive\right)\\ \;if\;Cluctch\;\left(n\right)\;is\;active \end{array}$$

where (N, β, θ, ϕ, ϕ), are the gear ratio between the sun gear and the planet gear, binary function, joint angular position at the current time, joint angular position at the activation time (*t*_*ON*_), and the backlash angle, respectively. The joint stiffness is the rate of change of the torque with respect to the angular deflection.

From the previous equations, the rendered torque (τ^Σ^) and the total stiffness (*K*_Σ_) can be derived as follows:

(5) $$\begin{array}{r} \tau^{\Sigma }=\sum_{0}^{n}N_{n}\beta_{n}\left(2^{n}\left(K_{0}\right)\;\left(\theta -\phi_{n}-\varphi \right)\right)\;,\;n\in \left\{0,1,2\right\} \end{array}$$

(6) $$K_{\Sigma }=\;\frac{\delta \tau^{\Sigma }}{\delta \theta }=\sum_{0}^{n}N_{n}\beta_{n}\left(2^{n}\left(K_{0}\right)\right)\;,\;n\in \{0,1,2\}$$

From Equation (5), it can be concluded that the involvement of each spring is independent from other spring. Hence, the level of stiffness can be altered at any position without the need of reverting to the initial equilibrium point. From Equation (6), it can be concluded that the joint stiffness is dependent on the number of stiffness-bits (*n*) and the base stiffness value (***K*****_0_**). This feature allows the scalability of the model in both the stiffness range and the realized number of stiffness values.

## The dynamic modeling of the BcVSA

Assuming the absence of backlash in gears, the dynamic equations of the system can be written in the following form

(7) $$\begin{array}{r} I_{m}{\ddot{\theta }}_{m}+B_{m}{\overset{\cdot }{\theta }}_{m}+NK_{eq}\left(\theta_{m}-\theta_{L}\right)=T_{in} \end{array}$$

(8) $$\begin{array}{r} I_{L}{\ddot{\theta }}_{L}+B_{L}{\overset{\cdot }{\theta }}_{L}+NK_{eq}\left(\theta_{L}-\theta_{m}\right)=T_{ext} \end{array}$$

Where:

*N*: is the gear ratio.

*K*_*eq*_: is the equivalent stiffness of BcVSA.

*I*_*m*_: is the motor inertia.

*I*_*L*_: is the load inertia.

*B*_*m*_: is the damping on the motor side.

*B*_*L*_: is the damping on the load side.

*T*_*in*_: is the motor torque.

*T*_*ext*_: is the load torque.

The dynamic model of our BcVSA can be represented by eight possible mathematical models. This is due to the fact that *K*_*eq*_ has eight possible values which depends on the status of clutches as listed in Table 1. This is the reason which motivated us to use Multiple MPC instead of using simple linear MPC as detailed in section Control Schemes. The main idea is to design a Multiple MPC which can incorporate all the possible mathematical models representation of BcVSA instead of using a simple linear MPC which can only handle one model. Consequently, the expected results for this approach is to increase the accuracy, the precision, and to reduce the uncertainty as much as possible. However, it is worth to mention that one representation of the dynamical model is not considered in this paper which is the one when all clutches are deactive since the system is decoupled completely in that case.

Thus, Equations (7, 8) can be written in the following form:

(9) $$\begin{array}{r} {\ddot{\theta }}_{m}=\frac{T_{in}-B_{m}{\overset{\cdot }{\theta }}_{m}+NK_{eq}\theta_{L}-NK_{eq}\theta_{m}}{I_{m}} \end{array}$$

(10) $$\begin{array}{r} {\ddot{\theta }}_{L}=\frac{T_{ext}-B_{L}{\overset{\cdot }{\theta }}_{L}-NK_{eq}\theta_{L}+NK_{eq}\theta_{m}}{I_{L}} \end{array}$$

Accordingly, seven state space representations can be derived. Due to space limitation, the major structure for the state space representation is shown in the following equations:

*q*_1_ = θ_*m*_, $q_{2}={\overset{\cdot }{\theta }}_{m}$, *q*_3_ = θ_*L*_, $q_{4}={\overset{\cdot }{\theta }}_{L}$

(11) $$\begin{array}{r} \overset{\cdot }{q}\left(t\right)=Aq\left(t\right)+Bu\left(t\right) \end{array}$$

(12) $$\begin{array}{r} y\left(t\right)=Cq\left(t\right)+Du\left(t\right) \end{array}$$

$q={[\begin{array}{c} q_{1}q_{2}q_{3}q_{4} \end{array}]}^{T}$: is known as the state vector.

$u={[\begin{array}{c} {\text{T}}_{in}{\text{T}}_{ext} \end{array}]}^{T}$: is known as the input vector.

$A=\left[\begin{array}{cccc} 0 & 1 & 0 & 0\\ -\frac{{\text{NK}}_{eq}}{I_{m}} & -\frac{{\text{B}}_{m}}{I_{m}} & \frac{{\text{NK}}_{eq}}{{\text{I}}_{m}} & 0\\ 0 & 0 & 0 & 1\\ \frac{{\text{NK}}_{eq}}{{\text{I}}_{\text{L}}} & 0 & -\frac{{\text{NK}}_{eq}}{{\text{I}}_{\text{L}}} & -\frac{{\text{B}}_{\text{L}}}{I_{L}} \end{array}\right],\;B=\left[\begin{array}{cc} 0 & 0\\ \frac{1}{{\text{I}}_{m}} & 0\\ 0 & 0\\ 0 & \frac{1}{{\text{I}}_{\text{L}}} \end{array}\right]$

**C**: is an identity matrix while it is called the output matrix.

**D**: is a zero matrix while it is called a feedforward matrix.

It is obvious this structure of the state space representation is fixed in principle but there is uncertainty in matrix ***A*** based on the value of *K*_*eq*_ i.e., based on the status of clutches. Nonetheless, there are two possible approaches that potentially can deal with this issue. The first approach in based on selecting a single controller that has the capability to deal with this kind of uncertainty e.g., *H*_∞_ controller. On the other hand, the second approach focuses on building multiple controllers where each one can deal with a specific mathematical model efficiently. In this paper, we implemented the second approach (see, section Control Schemes) as it is more inline to the dynamic model of our actuator. In this control approach, a selection criterion must be developed to select the suitable controller for each model. In our BcVSA, the switching between controllers depends on the status of active and inactive clutches.

## Control schemes

In this section, we present three possible controllers for controlling our BcVSA. The ultimate objective is to evaluate the performance of each controller and to select the most suitable one for the future experiments on our actuator. Each controller has its own pros and cons which mainly depend on the design requirements and criteria. In particular, we implemented the following controllers.

- Multiple MPC
- Multiple Explicit MPC
- Multiple Explicit MPC but with limited capability to calculate the sub-optimal solution instead of computing the optimal solution. In this paper, it is called the Approximated Multiple Explicit MPC.

The Multiple MPC composed of a multiple linear MPC (*MPC*_1_, …, *MPC*_*n*_). Nevertheless, each MPC works on a specific operating point i.e., it uses a specific state space representation. Based on that, the Multiple MPC has the capability to deal with several state representations to describe the behavior of the system efficiently. In other words, the Multiple MPC increases the range of the controller because it is providing (*n*) domain of attractions for (*n*) operating points. Furthermore, in each operating point, the system is linearized in case if it is a nonlinear system. Moreover, the uncertainty is also eliminated since the state space representation is known at each stage e.g. in our case the status of clutches. Apart from these pros of the Multiple MPC, it has a limitation in terms of computation load.

**Figure 3:**
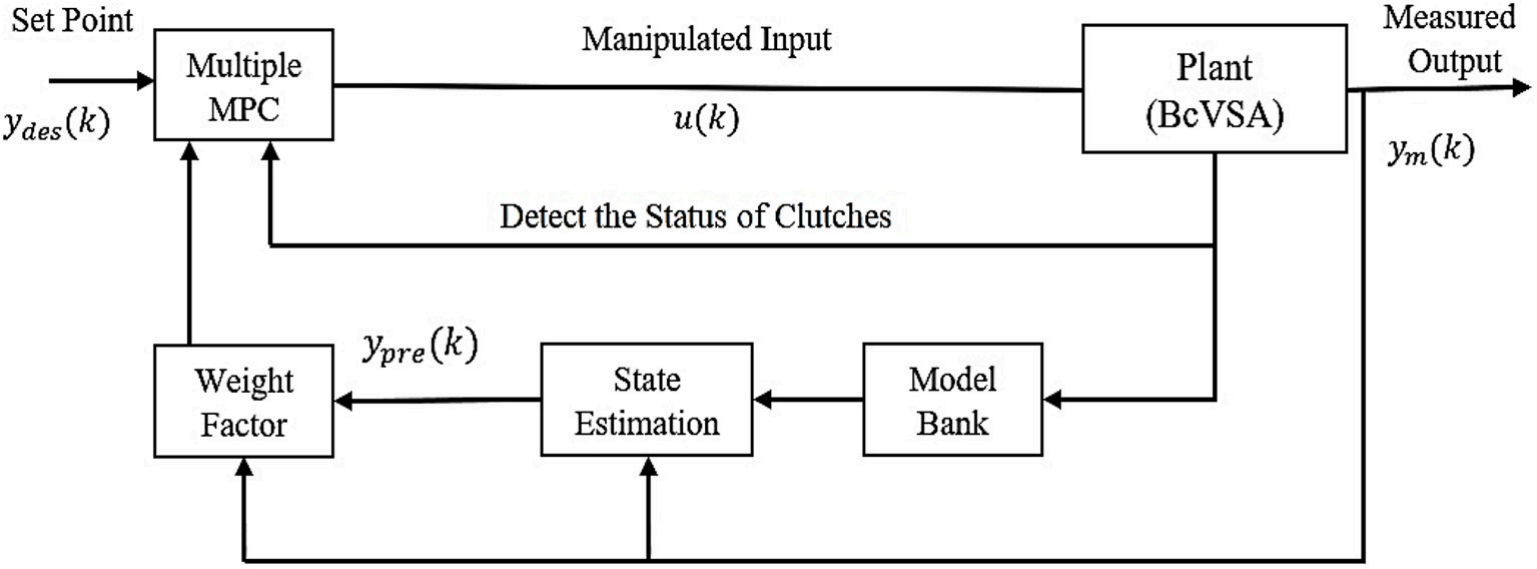
The control block diagram of Multiple MPC strategy.

This is due to the fact that every time, it needs to select the suitable linear MPC from the given set of Multiple MPC, then the selected MPC (e.g., *MPC*_1_) solves the constraint optimization problem that depends on the representation of the corresponding state of the system. For example, in our case, it depends on the status of the clutches While any change in the status of the clutches means it is required to select another linear MPC (e.g., *MPC*_2_) based on the new dynamic equations for the system at the second operating point. Then, *MPC*_2_ solves the second constraint optimization problem. The control block diagram of Multiple MPC is shown in Figure 3.

Consequently, it is expected this type of control may not be suitable for applications having fast dynamics e.g., robotics unless it is implemented on a high computational platform. For this reason, in literature, it's mainly used in the process applications or thermal applications. However, this hypothesis is planned to be tested in future on hardware prototype of the actuator and is out of the scope of the current paper's contribution.

In this research, the selected cost function (also known as the performance index) for the Multiple MPC is shown in Equation (13). The Matlab provides a toolbox for Model Predictive Control which we have used to control the BcVSA [32]:

(13) $$\begin{array}{r} \emptyset =\emptyset_{1}+\emptyset_{2}+\emptyset_{3} \end{array}$$

Where

(14) $$\begin{array}{rcl} \emptyset_{1} & = & \sum_{j=1}^{m}\sum_{i=1}^{p}{\left[\frac{w_{i,j}^{m}}{S_{j}^{y}}\left({y\;}_{des,j}\left(k+i|k\right)-y_{pre,j}\;\left(k+i|k\right)\right)\right]\;}^{2} \end{array}$$

(15) $$\begin{array}{rcl} \emptyset_{2} & = & \sum_{j=1}^{r}\sum_{i=0}^{p-1}{\left[\frac{w_{i,j}^{u}}{S_{j}^{u}}\left(u_{j}\left(k+i|k\right)-u_{j,nom.}\left(k+i|k\right)\right)\right]\;}^{2} \end{array}$$

(16) $$\begin{array}{rcl} \emptyset_{3} & = & \sum_{j=1}^{r}\sum_{i=0}^{p-1}{\left[\frac{w_{i,j}^{r}}{S_{j}^{u}}\left(u_{j}\left(k+i|k\right)-u_{j,nom.}\left(k+i-1|k\right)\right)\right]\;}^{2} \end{array}$$

Where:

k: is the current sample.

r: is the number of the inputs.

m: is the number of the outputs.

P: is the prediction horizon.

∅: is the total performance index for the constraint optimization problem.

∅_1_: is the performance index for minimizing the dynamic errors.

∅_2_: is the performance index to compute inputs near to the required nominal inputs (*u*_*nominal*_) or to minimize the inputs.

∅_3_: is the performance index for the change rates of the inputs.

*y*_*des,j*_(*k* + *i*|*k*): is the desired response for the j–th output at the i–th prediction horizon in the current sample (k).

*y*_*pre,j*_(*k* + *i*|*k*): is the predicted response for the j–th output at the i–th prediction horizon in the current sample (k).

$S_{j}^{y}$: is the scaling factor for the j–th output.

$S_{j}^{u}$: is the scaling factor for the j–th input.

$w_{i,j}^{m}$: is the weighting factor for the j–th output at the i–th prediction horizon.

$w_{i,j}^{u}$: is the weighting factor for the j–th input at the i–th prediction horizon.

*u*_*j*_(*k* + *i*|*k*): is the j–th input at the i–th prediction horizon in the current sample (k).

*u*_*j, nom*._(*k* + *i*|*k*): is the j–th nominal input at the i–th prediction horizon in the current sample (k).

The structure for the used discrete state space representation is shown in Equations (17, 18). This structure is used to predict the response in a finite horizon. Also, it is used as a constraint which the optimizer must respect to compute the optimal results. It is worth to mention that this structure is used in MPC Toolbox available in Matlab (2018). Furthermore, it is necessary to mention this structure is the same structure which is introduced in Equations (12, 13) but after converting it to the discrete domain, adding the capability to take into consideration the measured disturbance, and adding the capability of scaling the states.

(17) $$\begin{array}{rcl} q_{d}\left(k+1\right) & = & A_{d}q_{d}\left(k\right)+B_{d}S^{u}u_{d}\left(k\right)+B_{md}S^{\text{u}}v_{\text{d}}\left(k\right) \end{array}$$

(18) $$\begin{array}{l} y_{d}\left(k\right)={\left(S^{y}\right)}^{-1}C_{d}q_{d}\left(k\right)+{\left(S^{y}\right)}^{-1}D_{d}S^{u}u_{d}\left(k\right)\\ \;+{\left(S^{y}\right)}^{-1}D_{md}S^{u}v_{d}(k) \end{array}$$

Where:

***q***_***d***_ (k): the discrete state vector at the k–th sample.

***u***_***d***_ (*k*): the input signals at the k–th sample.

***v***_***d***_(*k*): the measured disturbance at the k–th sample.

***S***^***u***^: diagonal scaling input matrix.

***S***^***y***^: diagonal scaling output matrix.

***A***_***d***_: the discrete dynamic matrix.

***B***_***d***_: the discrete input matrix corresponds to the discrete states.

***B***_***md***_: the discrete input matrix corresponds to the measured disturbance

***y***_***d***_(k): the discrete output vector at the k–th sample.

***C***_***d***_: the discrete output matrix.

***D***_***d***_: the discrete feedforward matrix corresponds to the discrete inputs and discrete outputs.

***D***_***md***_: the discrete feedforward matrix corresponds to the discrete measured disturbance and discrete outputs.

While the used constraints in this project are shown in Equations (19, 20).

(19) $$\begin{array}{r} \frac{{u\left(i\right)}_{j,\;\min}}{S_{j}^{u}}\leq \frac{u_{j}\left(k+i|k\right)}{S_{j}^{u}}\leq \frac{{u\left(i\right)}_{j,\max}}{S_{j}^{u}} \end{array}$$

(20) $$\begin{array}{r} \frac{△{u\left(i\right)}_{j,\min}}{S_{j}^{u}}\leq \frac{△u_{j}\left(k+i|k\right)}{S_{j}^{u}}\leq \frac{{△u\left(i\right)}_{j,\max}}{S_{j}^{u}} \end{array}$$

Where:

*u*(*i*)_*j*, max_: is the maximum allowable torque for the j–th input torque at the prediction i.

*u*(*i*)_*j*, min_: is the minimum allowable torque for the j–th input torque at the prediction i.

△*u*(*i*)_*j*, min_: is the minimum allowable change of rate for the j–th input torque at the prediction i.

△*u*(*i*)_*j*, max_: is the maximum allowable change of rate for the j–th input torque at the prediction i.

Finally, the total performance index with the selected constraints and the seven mathematical models are rewritten in the form of seven QP problems as shown in Equarion (21).

(21) $$\begin{array}{r} Min_{x}\left[\frac{1}{2}q^{T}Hq+f^{T}q\right] \end{array}$$

sub to: $\bar{A}q\geq \bar{b}$

where: H is the Hessian matrix.

$\bar{A}$: is the matrix linear constraints

$\bar{b}$ and $\bar{f}$: are vectors.

The second controller is the Multiple Explicit MPC. This controller consists of several Explicit MPC controllers (Explicit *MPC*_1_, …, Explicit *MPC*_*n*_). Furthermore, this controller is used because it has the same advantages like the Multiple MPC but with an interesting feature that is related to a better capability to work in the real-time environment. This is due to the fact that the Explicit MPC solves the optimization problem in the off-line phase and the results are save in a specific look-up table (also known as polyhedral regions (see **Figure 7**) to compute the control action C(k) by using the affine function instead of solving the constraint optimization problem in the online framework (Matlab, 2018). This is achieved by adding constraints on the range of the state, the outputs, the measures disturbances if there is any to reduce the number of the polyhedral regions (see Equations 22, 23). Some other methods based on the states feedback control with restriction are presented in José de Jesús (2018), Pan et al. (2016), and José de Jesús et al. (2017).

Finally, the control action u(k) is computed by detecting the states, the outputs, and the measured disturbances if they are existing as shown in Equation (24).

(22) $$\begin{array}{r} q_{l.b.}\leq q\left(k\right)\leq q_{u.b}\; \end{array}$$

(23) $$\begin{array}{r} H_{i}q\left(k\right)\leq K_{i},\;i=1:n_{r} \end{array}$$

(24) $$\begin{array}{r} u\left(k\right)=F_{i}q\left(k\right)+G_{i},\;i=1:n_{r} \end{array}$$

Where:

***q***_***l***.***b***._: lower bound for the state vector.

***q***_***u***.***b***._: upper bound for the state vector.

*H*_*i*_, *K*_*i*_, *F*_*i*_, and, *G*_*i*_ are special constants for each polyhedral region.

Nonetheless, the inactive Explicit MPC controllers do not provide any input to the system from their database because these inputs are not the optimal inputs but these are calculated continuously through the state estimation. This is due to the fact that the inputs must be ready in case the dynamics of the system changes and the corresponding Explicit MPC is being activated (As is it happens each time the status of the clutches of our system changes) (Matlab, 2018).

**Figure 4:**
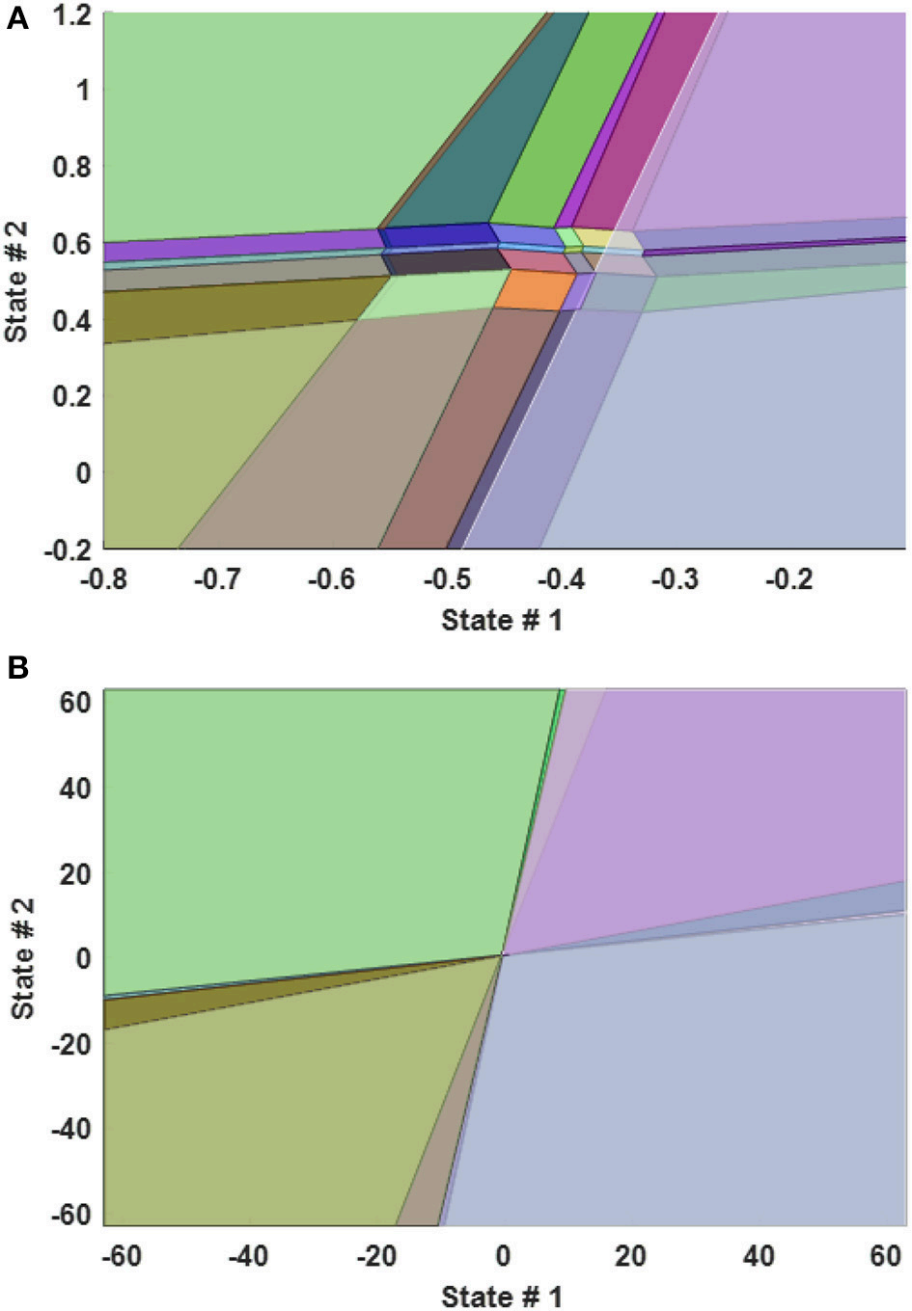
2-D Plot of Explicit MPC Polyhedral Regions between the state 1 and state 2 when the other states are fixed. **(A)** zoomed plot **(B)** full range plot.

By using this approach, the system not only has the capability to work on seven different operating points of our dynamic model but even reduced the computation time dramatically. The third control approach focuses on utilizing Approximated Multiple Explicit MPC but with requesting from each Explicit controller to calculate the sub-optimal input. This is achieved by using the available command in MATLAB. However, this approach will reduce the polyhedral regions by combining the small regions (see Figure 4) to the closer regions, which reduces the required size for the memory, but it provides the sub-optimal solution to the plant (Matlab, 2018). The methodology of this approximation is based on the criterion that if the radius of any region is smaller than a specific value of the Chebychev radius. Then it's combined with the closer region as an approximation solution.

## Numerical results

In this section, we apply previously introduced controllers on the dynamic model of our BcVSA and perform numerical simulations. In particular, we evaluate their performance on tracking the desired responses and the capability of each controller to deal with a measured disturbance. In this regard, we performed total six case studies, out of which, five case studies focused on the evaluation of the performance of each Multiple MPC controller under different applied conditions while the sixth case study compares the performance of the system under Multiple MPC and other two types of controller (LQR, CTC). In all cases, the active status (engage) of clutch refers to an active stiffness bit (“1”) while inactive or de-active status (disengage) represents an inactive stiffness bit (“0”).

The first two case studies focus on testing the capability of all controllers to track the desired angular positions under the conditions of both changing and unchanging the status of the clutches. In these two cases, we don't evaluate the ability of the controllers to reject a measured disturbance input. On the other hand, in next three case studies, we focused on testing the robustness of each controller under the effect of different types of measured disturbances. The disturbances were considered in the form of sinewave, random, and step, under the effect of the both changing and unchanging the status of clutches. Finally, the last case study focused on comparing the performance of the system when the following controllers are used: Multiple MPC, Multiple Explicit MPC, Approximated Explicit MPC, Computed Torque Control (CTC), and Linear Quadratic Regulator (LQR).

In all the simulation results, some of the dynamic parameters are assumed while the others are known because they are selected in the design phase. The assumed parameters are the inertia (*I*) and damping (*B*) at both motor (*m*) and load side (*L*). The next natural step of the project will be to identify the assumed parameters using the hardware prototype of the BcVSA and confirm the simulation results with the actual identified parameters.

### Case study 1:

In the first case study, we make a comparison between three types of previously introduced controllers (i.e., Multiple MPC, Multiple Explicit MPC, and Approximated Multiple Explicit MPC). We designed and built three tracking systems based on these controllers. Two cubic polynomial trajectories are used as desired signals that are: *q*_1_−_*des*_ and *q*_3_−_*des*_ i.e., θ_*m*_−_*des*_ and θ_*L*_−_*des*_ respectively.

Accordingly, the following design parameters are selected: the sampling time (*T*_*s*_ = 1 ms), the prediction horizon (*P* = 20), the control horizon (*C* = 2), the weightings for the outputs ($w_{j}^{m}$ = 3000), the weighting for the change rates of inputs ($w_{j}^{du}$ = 0.1). While the selected constraints used are the maximum and minimum allowable motor torque (*T* ≤ ±60) and to the rate of change of the torque inputs (Δ*T* ≤ ±30). All the other design parameters used are the default options provided by Matlab. Moreover, for the sake of performance comparison, same parameters are used for each class of MPC. It is worth to note that Multiple MPC uses these design parameters to solve the optimization problem in the online framework while the other two types (Multiple Explicit MPC and the Approximated Multiple Explicit MPC) deploy them to solve the optimization problem in the offline phase. In this regard, it is required for the controllers working on the off-line phase to specify the ranges for the states, the inputs, and the outputs to limit the polyhedral regions to a finite number. The parameters' region can be specified based on the understanding of the dynamical model for the system and the required dynamical behavior e.g., the selected range for the states and for the reference signals can be bounded between (±20^*^ π) and the selected range for the inputs (torque) can be bounded between ±60 N.m. (based on the capability of the actuators). Consequently, this test checks the adequacy of each controller to track the desired responses in the case of Bit 2^2^ clutch is active i.e. the *K*_*eq*_ = 4 K_0_.

It is worth to mention that the selected criterion to compute the sub-optimal solution is focused on combining the small region if it has a Chebychev radius smaller than 0.001 with a bigger region as an approximation solution to reduce the memory size and to approximate the optimal solution. Table 2 shows the comparison between the multiple explicit MPC and the approximated multiple explicit MPC based on the status of clutches and the corresponding polyhedral regions.

**Table 2:** Comparision between the multiple explicit MPC and the approximated multiple explicit MPC based on the status of clutches and the polyhedral regions.

**Stiffness level**	Number of polyhedral regions	
	**Multiple explicit MPC**	**Approximated multiple explicit MPC**
K_0_	399	400
2 K_0_	405	406
3 K_0_	399	422
4 K_0_	383	418
5 K_0_	387	421
6 K_0_	387	421
7 K_0_	379	417

**Figure 5:**
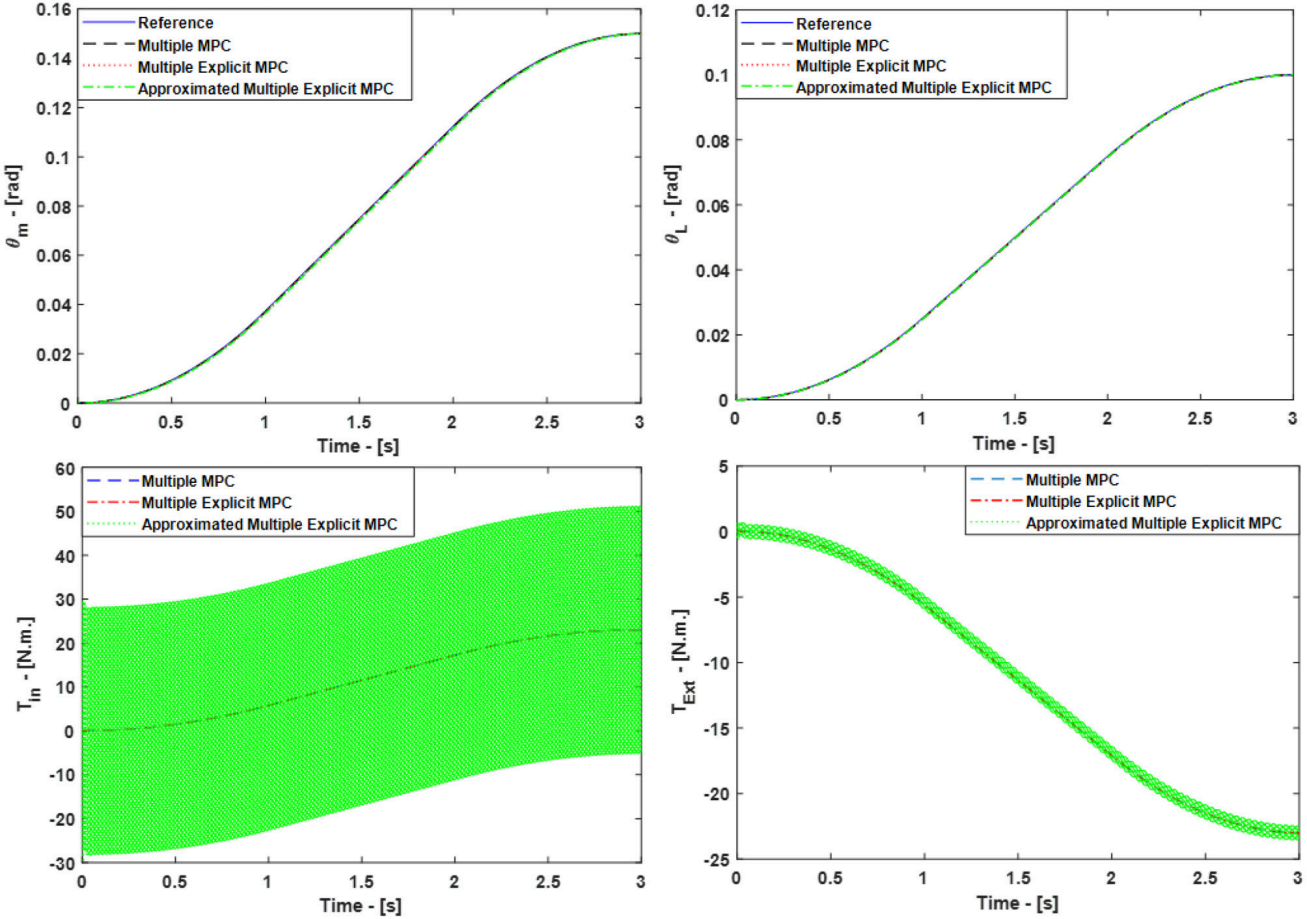
Case Study 1: On top, comparison for the reference command (θ_*m*_) and (θ_*L*_), On bottom, comparison for the input torque (*T*_*in*_) and (*T*_*Ext*_), the responses of Multiple MPC, Multiple Explicit MPC, and approximated Multiple Explicit MPC.

As a result, all three controllers were able to track the desired reference trajectories as shown in Figure 5, top. Moreover, the inputs *T*_*in*_ and *T*_*Ext*_ used in this case are shown in **Figure 8**, bottom. It is observed that similar results were achieved in the case of Multiple MPC and Multiple Explicit MPC and these results were expected because the major difference between them is the fact that the Multiple MPC solves the optimization problem during on-line phase while the other solve it in the off-line phase. On the other hand the, the Approximated Multiple Explicit MPC has the ability to track the desired references but it consumes more energy as shown in Figure 5.

The main reason of this is the fact that, under the provided conditions, Multiple Explicit MPC calculated the optimal results and memorized the results into 418 polyhedral regions while the approximated Multiple Explicit MPC calculated the sub-optimal results and memorized the results into 383 polyhedral regions as shown Table 2. As the matter of fact, the approximated Multiple Explicit MPC uses a methodology to compute the sub-optimal results by combining the small polyhedral regions to the bigger polyhedral regions as an approximation and uses the affine functions for the bigger (combined) regions.

### Case study 2:

In the second case study, we used the same design parameters e.g., weighting factors, constraints, and ranges for the controllers as previously used in case study 1. While, in this scenario, we tested the ability of each controller to control the BcVSA in the conditions of changing the equivalent stiffness while the system is running. The final time selected for the cubic trajectory-planning algorithm was 3 s. During this total interval, we applied different stiffness level (*K*_*eq*_) by changing the status of the clutches in different sub-time interval. In particular,

- during the first second ([0≤t<1]) the status of all clutches are activated i.e. (*K*_*eq*_ = 7 K_0_).
- While the status of the Bit 2^0^ clutch is deactivated i.e. (*K*_*eq*_ = 6 K_0_) in the second interval ([1≤t<2]) second.
- Finally, in the third interval ([2≤t≤3]) second, the clutches of Bit 2^0^ and Bit 2^2^ are activated and the one of Bit 2^1^ deactivated i.e., (*K*_*eq*_ = 5 K_0_).

**Figure 6:**
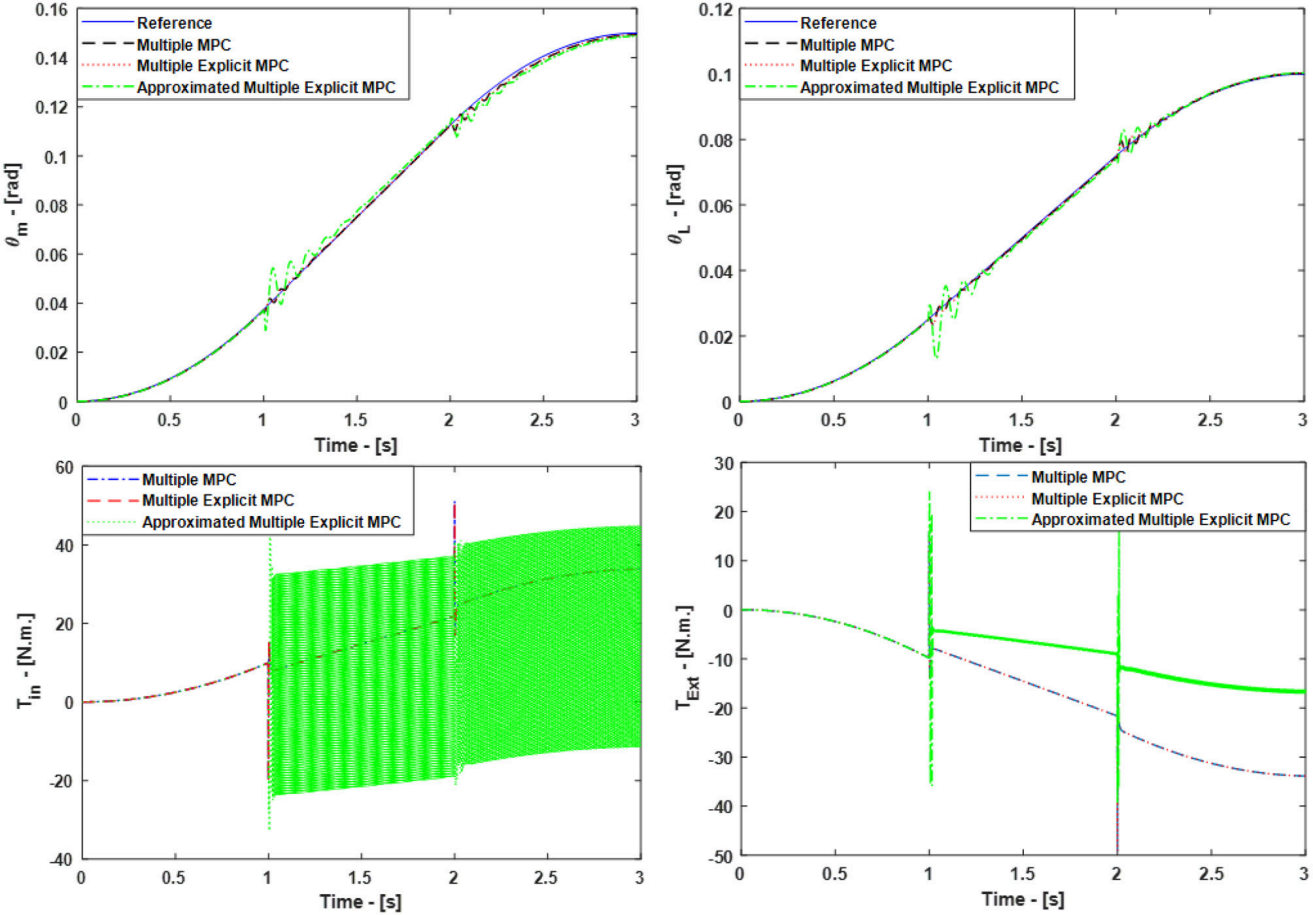
Case Study 2: On top, comparison for the reference command (θ_*m*_) and (θ_*L*_), On bottom, comparison for the input torque (*T*_*in*_) and (*T*_*Ext*_), the responses of Multiple MPC, Multiple Explicit MPC, and approximated Multiple Explicit MPC.

The simulated responses for the Multiple MPC, the Multiple Explicit MPC, and the approximated Multiple Explicit MPC under the above listed conditions are shown in **Figure 9**, top. While the input torques for each case are shown in Figure 6, bottom.

The results show the ability of all controller to work under these applied conditions. But it is observed the Multiple Explicit MPC consumes more energy to perform the required tasks.

### Case study 3:

**Figure 7:**
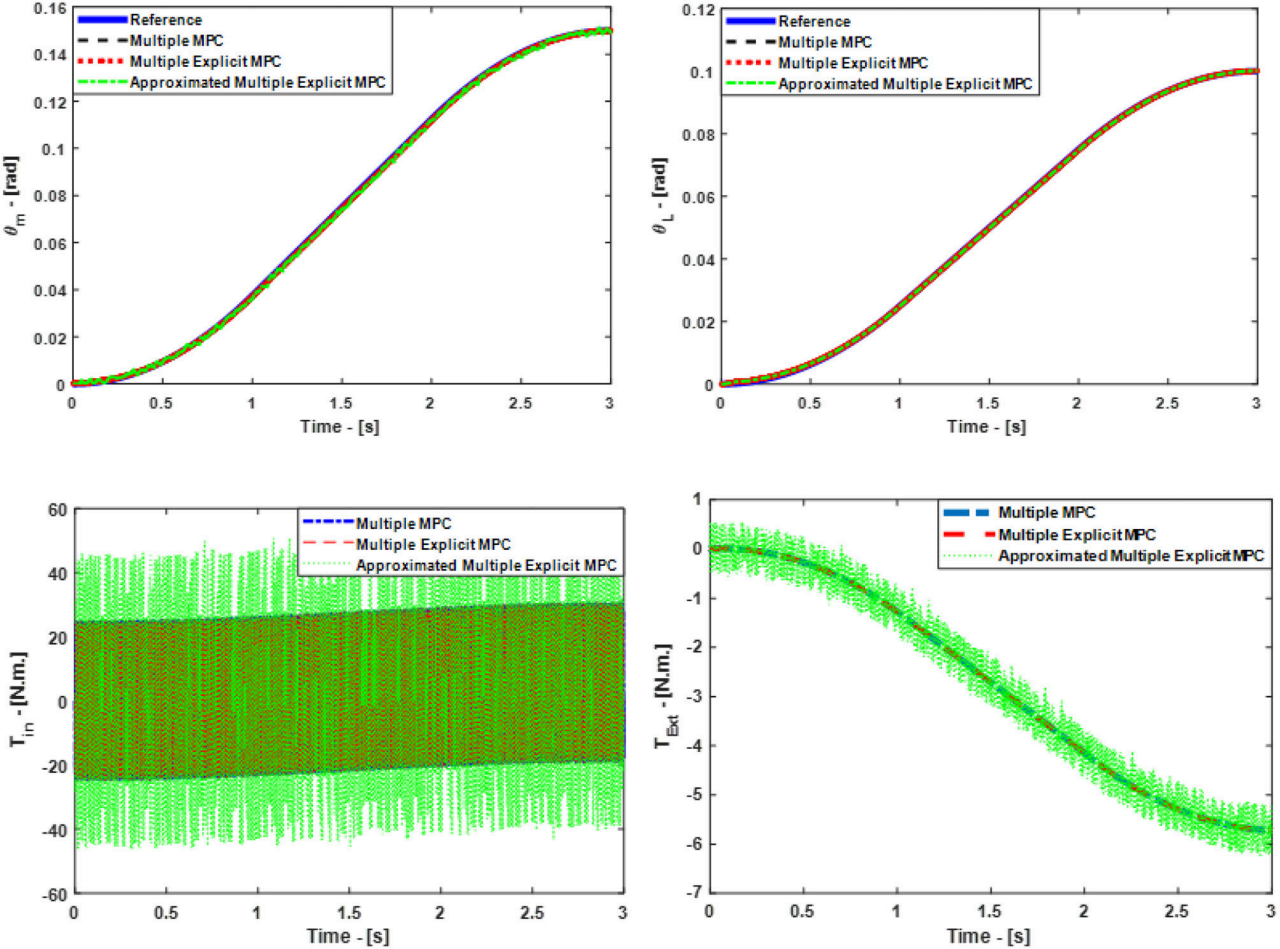
Case Study 3: On top, comparison for the reference command (θ_*m*_) and (θ_*L*_), On bottom, comparison for the input torque (*T*_*in*_) and (*T*_*Ext*_), the responses of Multiple MPC, Multiple Explicit MPC, and approximated Multiple Explicit MPC.

In this case study, some of the control parameters are changed to make the control problem even more challenging. The updated parameters are: the prediction horizon (*P* = 40), the control horizon (*C* = 2). While weighting factors are same as before. But, this time, we activated the option to measure the disturbance for all the controllers see Equations (17, 18). Furthermore, the selected constraints: are the maximum and minimum allowable motor torque (*T* ≤ ±60 N.M) and the allowable rate of change of the torque inputs (Δ*T* ≤ ±30). However, it is necessary in the case of the Multiple Explicit MPC and in the case of the Approximated Multiple Explicit MPC to specify the range of the disturbance. Consequently, the selected range for the measured disturbance is (*T*_*dist*._ ≤ ±40) also it is assumed this disturbance effect is on the motor side. Accordingly, the selected input disturbance in this scenario is a sine wave with an amplitude 25 and it has 1000 rad/s as a force frequency. Figure 7, top presents the simulated responses for θ_*m*_ and θ_*L*_ respectively under the above mentioned applied conditions. While the input signals are shown in Figure 7, bottom. Finally, it is worth to mention in this scenario the active clutch is only Bit 2^0^ i.e., (*K*_*eq*_ = K_0_).

It is obvious all controllers succeeded to reject the disturbance efficiently in principle but the steady state error in the case of Approximated Multiple Explicit MPC more as compared to others. In other words, the Approximated Multiple Explicit MPC controller relatively faces problems in tracking the desired responses because it consumes more energy as comparison to the others as shown in Figure 7.

### Case study 4:

**Figure 8:**
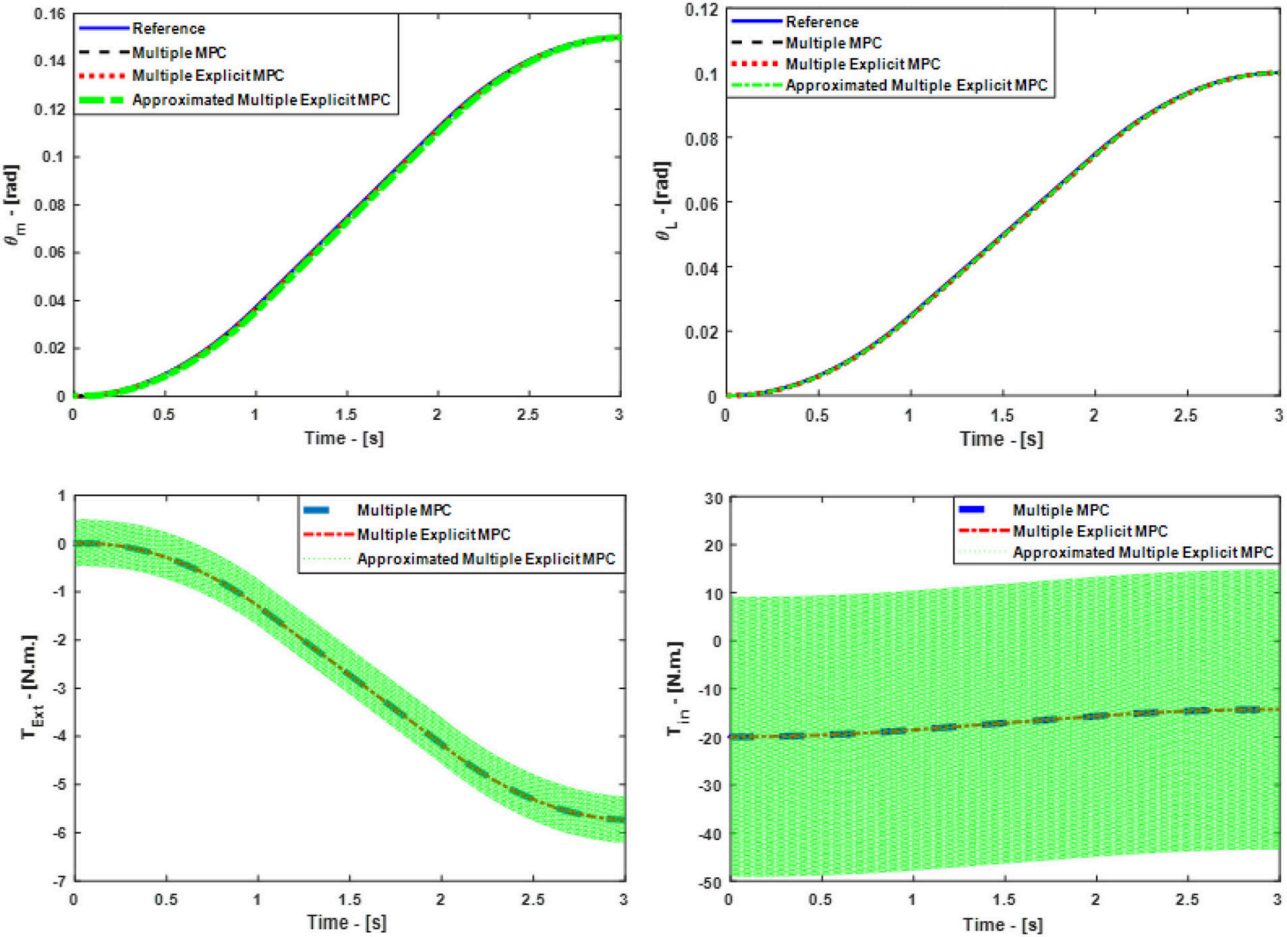
Case Study 4: On top, comparison for the reference command (θ_*m*_) and (θ_*L*_), On bottom, comparison for the input torque (*T*_*in*_) and (*T*_*Ext*_), the responses of Multiple MPC, Multiple Explicit MPC, and approximated Multiple Explicit MPC.

This scenario focuses on testing the capability of all the controllers to reject a disturbance in the form of step wave which is effected on the motor side (*T*_*dist*_ = 20 N.m.). Furthmore, the Bit 2^0^ clutch is activated based on that, the equivalent stiffness in the model is equal to K_0_. It is worth to mention the controllers' parameters are similar to the parameters which are used in the third case study. Consequently, a comparison between all the controllers is made based on: the ability to track the desired references, the robustness, and the consumed torque (energy) to perform the tasks. As a result, Figure 8, top shows the ability of all the controllers to track the desired references in principle. But, it is observed the steady-state error is a little bit more in the case of Approximated Multiple Explicit MPC as compared to the other controllers. In addition, the Approximated Multiple Explicit MPC consumed more input torque to follow the reference trajectories and to reject the disturbance input at the same time (see Figure 8, bottom). The possible reason is because this controller calculates the sub-optimal solutions which means it can be expected that the desired results will be achieved with comparatively more input i.e., this controller consumes more energy from the control effort point of view. Moreover, the robustness of this controller is less than as compared to others because there are several small polyhedral regions which are combined with others in case the Chebychev radius is less than 0.001 as a sub-optimal solution. It is worth to mention the selection of the Chebychev radius is a critical point because if this radius is increased that means the number of the approximated small regions are increased too i.e., the approximated sub-optimal results will be far away as compared to the optimal results especially under the effect of such disturbance.

### Case study 5:

**Figure 9:**
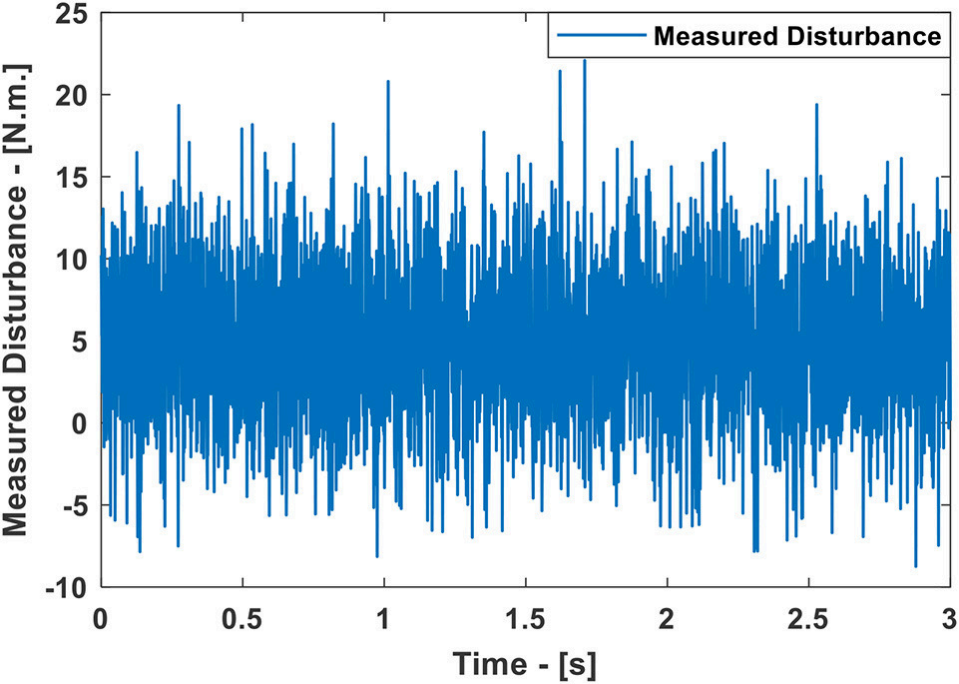
The measured disturbance in the Case Study 5.

This scenario uses the same control parameters as introduced in the case study 3. But in the current scenario, there are two major differences: First, a random input disturbance is applied and second, different status of the active clutches is considered as compared to previous one. The selected parameters for the random disturbance is equal to 5 N.m. as a mean value having the variance equal to 20 (see Figure 9). The final time selected for the cubic trajectory planning algorithm is 3 s. Under these conditions,

- during the first second [0≤t<1] the status of all clutches are activated i.e. (*K*_*eq*_ = 7 K_0_).
- While the status of the Bit 2^1^ clutch is activated i.e., (*K*_*eq*_ = 2 K_0_) in the second interval [1≤t<2] second.
- Finally, in the interval [2≤t≤3] seconds, the Bit 2^0^ and the Bit 2^1^ clutches are activated while the Bit 2^2^ clutch is deactivated i.e., (*K*_*eq*_ = 3 K_0_).

**Figure 10:**
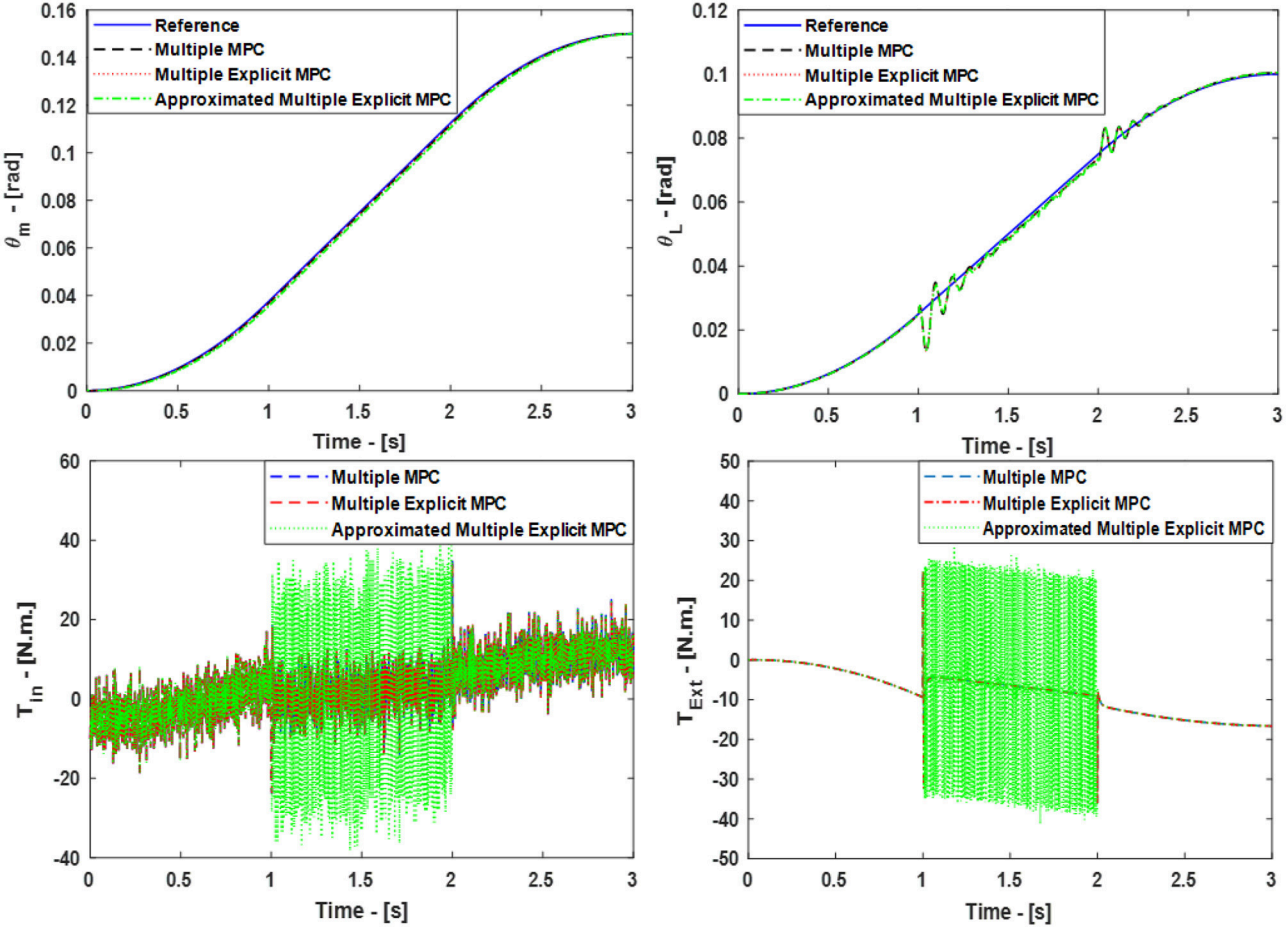
Case Study 5: On top, comparison for the reference command (θ_*m*_) and (θ_*L*_), On bottom, comparison for the input torque (*T*_*in*_) and (*T*_*Ext*_), the responses of Multiple MPC, Multiple Explicit MPC, and approximated Multiple Explicit MPC.

The simulated responses for the Multiple MPC, the Multiple Explicit MPC, the Approximated Multiple Explicit MPC under the above-mentioned conditions are shown in Figure 10, top. While the torque inputs are shown in Figure 10, bottom. Consequently, it is obvious all the controllers performed well in rejecting disturbance and under the situation of changing the clutches' status.

### Case study 6:

**Figure 11:**
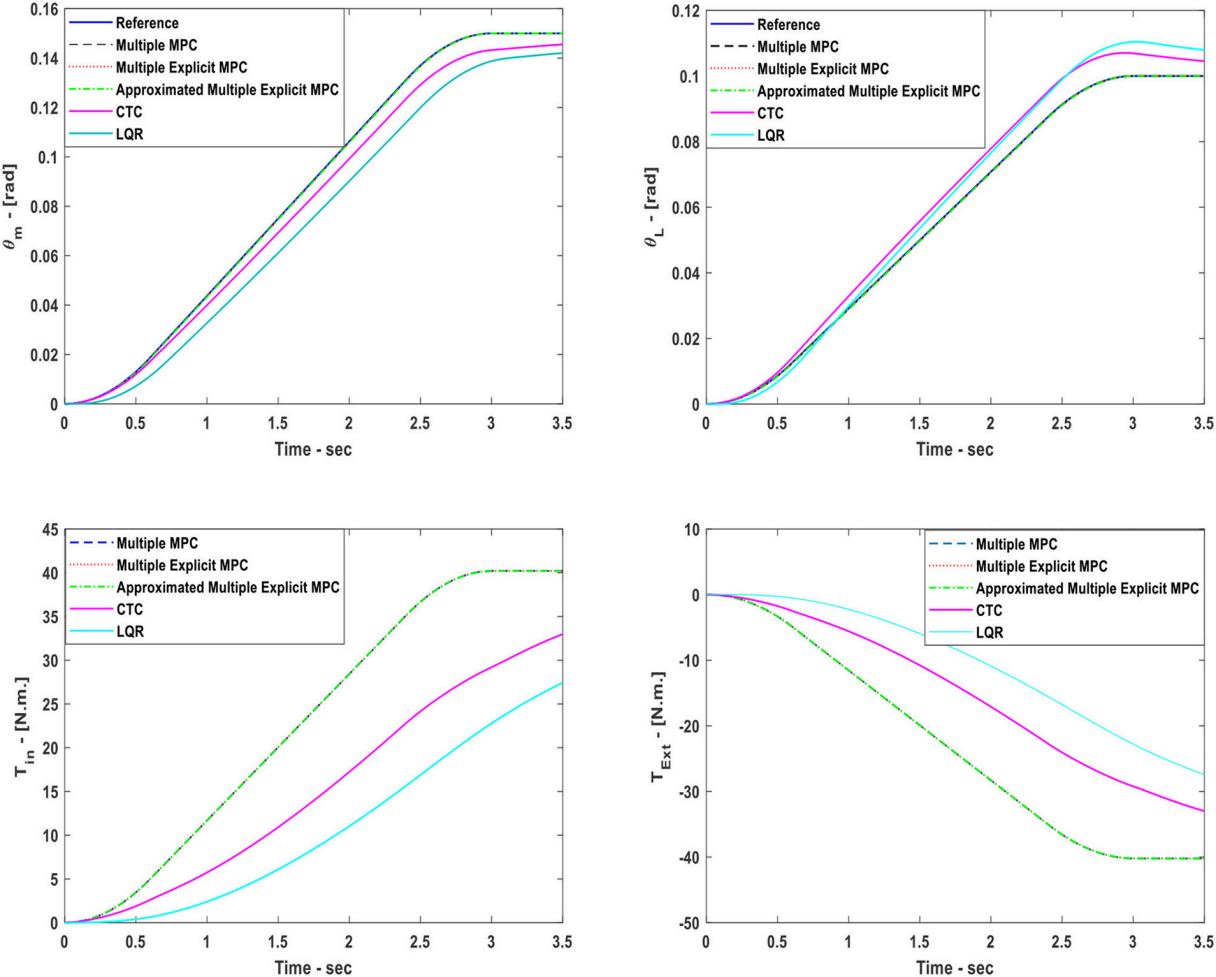
Case Study 6: On top, comparison for the reference command (θ_*m*_) and (θ_*L*_), On bottom, comparison for the input torque (*T*_*in*_) and (*T*_*Ext*_), the responses of Multiple MPC, Multiple Explicit MPC, and approximated Multiple Explicit MPC, CTC, and LQR, are shown.

This case study focuses on evaluating the performance of the system under MPC based controllers with LQR and CTC controllers. In the current scenario, two tracking system have been built to track the desired commands (θ_*m*_, θ_*L*_). The first tracking system uses the Computed Torque Control (CTC) method to perform the required tasks. While the second tracking system uses the Linear Quadratic Regulator (LQR) method to achieve the goals. In this scenario, the status of all clutches are activated i.e., (*K*_*eq*_ = 7 K_0_). As a result, all three MPC controllers were able to track the desired reference trajectories as shown in Figure 11, top, with steady state error equal to zero and with a very small settling time. Although, the CTC, and the LQR were also able to track the desired trajectories but with slower responses. Moreover, the inputs *T*_*in*_ and *T*_*Ext*_ used in this case are shown in Figure 11, bottom.

## Conclusions and future work

In this paper, the concept of a novel active variable stiffness actuator is introduced as a bench-test for further development toward the compliant manipulator. In particular, we presented the working principle, stiffness modeling and control of a novel Binary-Controlled Variable Stiffness Actuator (BcVSA). The BcVSA is the proof of concept of the active revolute joint with the variable recruitment of series-parallel elastic elements. We developed three controllers to control the BcVSA and evaluated the performance of each controller. This study was necessary to select the most suitable one for the future experiments on our actuator. This paper also tests the robustness of each control under several conditions e.g., in the case of presence of measured disturbance or/and in the case of changing the status of clutches. As a result, the Multiple MPC and the Multiple Explicit MPC have better performance from the perspective of robustness, accuracy, and the energy consumption as compared to the Approximated Multiple Explicit MPC. But it is expected the Approximated Multiple Explicit may have the advantage in terms of its capability to work in real-time i.e., it has less computation load. This is due to the fact that memorizes the data in the polyhedral regions and it calculates the sub-optimal solution only. Anyway, this hypothesis is not tested yet and is out of scope of this paper. We also performed the comparison of Multiple MPC based controllers with the LQR and CTC. In future, we are aiming to perform the experiments on the prototype of the actuator which is under the process of manufacturing. We will confirm all the simulation results with the experimental oneson the hardware. Moreover, we will evaluate the performance of each controller to check its capability to work in real time environment.

## Author contributions

IH contributed in actuator CAD design, dynamic modeling, control, and writing. AA contributed in the dynamic modeling, control and writing. MA contributed in the design, stiffness, and dynamic modeling and writing. LS contributed in overall leading this research, guidance and paper proof reading/correction. DG contributed in the research idea, leading the overall research, writing and paper proof reading/correction.

### Conflict of interest statement

The authors declare that the research was conducted in the absence of any commercial or financial relationships that could be construed as a potential conflict of interest.
